# Dangers of Oratory

**Published:** 1858-03

**Authors:** 


### DANGERS OF ORATORY.

AMONG the greatest orators of the last hundred years, there are many who have gone down to a drunkard's degradation and a drunkard's grave. Into the kennel, men of might have gone from the editorial sanctum, from the halls of legislation, from the forum, from the bar, from the stage, and from the pulpit itself, as witness—but we spare the dead, in fact, or in effect—any man of extensive observation can count up for himself. We can make use of the lesson, without harrowing up the feelings of the living.

This terrible vice of drunkenness is fallen into by the slowest degrees, by that class of young men who are considered "good at making a speech." Such soft names are sometimes given to liquor-drinking, that the designation itself is a lure—it at least is not a scare-crow. "To steady the nerves," "to wet the whistle," and the like.

Some persons "take a drink" before making a speech; others, during the time; a third class prefer taking it afterwards, which is certainly the safest. One of the greatest of modern orators arose to address an assembly in this city, in our hearing, but was so drunk that he not only had not the full command of his tongue and lips, but had to hold up himself by the support of the corner of a table. He had miscalculated!—not as to the quantity of liquor, or as to the time it would take to begin to affect him—he had doubtless made the calculation so often, and had gone through the "operation" so repeatedly, as to feel perfectly at home; the error was in his predecessor being too dull, and, as a consequence, too long, and the great man missed fire. EDMUND KEAN, the great player, never drank during a performance, but *always* after it, and drink killed him, before his time. KNOX, a celebrated English actor, required two quarts of brandy to enable him to go through, properly, with one of his great characters. And it was said of big JOHN REEVE, that he required a still greater quantity.

It is always safest and best for a man to rely on his own resources. He may not be so great "on occasion," but he will be more uniformly great, without the risk of disgracing himself and putting his friends and admirers to the blush.

We have known some clergymen, to whom a cup of tea or coffee was a necessity, in connection with a public effort, and we have seen their great light go out in the wine cup.

There is a kind of vacuum in mind and body, a goneness, a wanting of some stay, after a public discourse. The best and safest method, *in the long run*, is to retire to one's room, walk back and forth, read the shortest items of the latest newspaper, recline on a sofa, or play with a child, either or all of these, or alternating. In this way, they will be safely *let down* to their ordinary level.

As to clergymen who have in a few hours to make another effort, say on the Sabbath day, we advise them by all means to avoid taking a regular meal. Be content with one or two apples, or oranges, or crackers, or a piece of bread and butter.

Not long ago, we heard one of the most eminent, useful, and admired clergymen, a Western man, say, on being invited to a "Sunday dinner," after one of his most efficient discourses in this city, "*I never eat between sermons.*" He was the picture of health, and a pattern of pulpit oratory. Showing that he, at least, could be good and great, without saturating the carnal mass with meats and wine at the "Sunday dinner" table.

If church-goers would but "go half way" in this same direction, our Sunday afternoon discourses would not be so often wasted on unconscious ears. "The Sunday dinner" nullifies about one-half of a minister's labors. It is physiologically demonstrable.

